# Specific Therapy of Olfactory Disorders in COVID-19 Patients is Essential for the Prevention of Long-term Dysfunction

**DOI:** 10.1007/s12070-021-02818-z

**Published:** 2021-08-22

**Authors:** Luigi Angelo Vaira, Jerome R. Lechien, Stefano Dore, Riccardo Boccaletti, Sven Saussez, Giacomo De Riu

**Affiliations:** 1 Maxillofacial Surgery Unit, Department of Medical, Surgical and Experimental Sciences, University of Sassari, Viale San Pietro 10, 07100 Sassari, Italy; 2 Biomedical Science Department, PhD School of Biomedical Science, University of Sassari, Sassari, Italy; 3 COVID-19 Task Force of the Young-Otolaryngologists of the International Federations of Oto-Rhino-Laryngological Societies (YO-IFOS), Paris, France; 4 Department of Human and Experimental Oncology, Faculty of Medicine UMONS Research Institute for Health Sciences and Technology, University of Mons (UMons), Mons, Belgium; 5 Ophthalmology Operative Unit, Department of Medical, Surgical and Experimental Sciences, University of Sassari, Sassari, Italy; 6 Neurosurgery Operative Unit, University Hospital of Sassari, Sassari, Italy

**Keywords:** Covid-19, Anosmia, Smell disorders, Corticosteroids, Coronavirus, SARS-CoV-2, Therapy

Dear Editor,

We enjoyed reading the article by Borah et al. [1], recently published in your journal. The authors, starting from an extensive analysis of the otolaryngological symptoms of 2000 patients with a confirmed diagnosis of coronavirus disease 2019 (COVID-19), try to provide some general indications on the management of these patients. In this series of patients, a significant prevalence of olfactory and gustatory disorders was reported and, on this basis, the authors rightly suggest the opportunity to identify specific therapies for patients presenting these problems. The authors' intent must be commended and we would like to enrich the debate raised by this article with some information that emerged from the experience we have gained in recent months on olfactory disorders in COVID-19 patients.

First, the need to implement specific therapies is confirmed by the fact that the first prospective studies with long-term follow-up are finding the persistence of severe olfactory disturbances in 5.5–11% of patients 6 months after infection [2, 3]. Such a high frequency, given the high prevalence of infection in the general population, means that in the near future we will have a huge number of patients with long-term morbidity seeking assistance.

The first pathological studies in patients with long-term COVID-19 related anosmia revealed an inflammatory-based destruction of the olfactory epithelium (OE) with involvement of the basal cells and loss of the regenerative potential of the OE [4]. Although in most patients this inflammation allows the regeneration of the OE and the recovery of the sense of smell within a few weeks, in some cases the inflammatory process can persist even after the resolution of the infection, progressively and irreparably damaging the OE. Unfortunately, the risk factors for the development of a long-term olfactory disorder have not yet been identified.

However, these recent pathogenic findings have provided the rationale for the use of systemic and local corticosteroids for the treatment of persistent COVID-19 related olfactory disorders [5]. This specific therapy is all the more effective the earlier it is started. Considering that the use of corticosteroids in patients with mild and moderate COVID-19, we believe it is indicated to postpone the initiation of therapy after the nasopharyngeal swab become negative. Future studies will be needed to establish safety and the opportunity of starting corticosteroid therapy earlier.
